# Build the virtual cell with artificial intelligence: a perspective for cancer research

**DOI:** 10.1186/s40779-025-00591-6

**Published:** 2025-01-27

**Authors:** Tao Yang, Yuan-Yi Wang, Fei Ma, Bing-He Xu, Hai-Li Qian

**Affiliations:** 1 Department of Medical Oncology, National Cancer Center/National Clinical Research Center for Cancer/Cancer Hospital, Chinese Academy of Medical Sciences and Peking Union Medical College, Beijing, 100021 China; 2 State Key Laboratory of Molecular Oncology, National Cancer Center/National Clinical Research Center for Cancer/Cancer Hospital, Chinese Academy of Medical Sciences and Peking Union Medical College, Beijing, 100021 China; 3 National Cancer Center/Cancer Hospital, Chinese Academy of Medical Sciences and Peking Union Medical College, Beijing, 100021 China

**Keywords:** Virtual cell, Artificial intelligence, Cancer research, Multi-omics

Dear Editor,

The cell, as the fundamental unit of life, presents core challenges in biological research due to its inherent complexity and dynamic properties. A recent perspective published in *Cell* [1] and an out-of-box article in *The Innovation Life* [2] outlined our visions for constructing artificial intelligence virtual cells (AIVCs). Unlike other diseases, cancer is fundamentally a cellular disease. While other disciplines focus on pathophysiological changes at the system or organ level, cancer uniquely begins with abnormal proliferation triggered by genomic instability at the cellular level, subsequently inducing local microenvironment remodeling. This “bottom-up” pathogenic pattern makes oncology particularly amenable to investigation through AIVC technology. The rapid advancement of AI provides novel pathways for learning biological patterns and processes directly from data, without relying on predetermined rules or human annotation. Against this backdrop, this paper explores the transformative potential of AIVC technology from the perspective of cancer research.

## Definition of AIVCs

AIVCs were initially conceived as comprehensive digital twin physical models that would integrate and generalize cross-scale, multi-modal observational data through explicitly defined mathematical methods or AI technologies, enabling computational manipulation and observation of real-time simulated cellular molecular processes [3, 4]. However, cells are extraordinarily complex dynamic adaptive systems characterized by countless nonlinear molecular interactions, where small input changes can lead to intricate output variations. This complexity is particularly critical for cancer cells, where minor perturbations in cellular phenotypes can directly impact clinical decisions. For instance, in breast cancer patients routinely receiving endocrine therapy, single-point mutations in *PIK3CA* (phosphatidylinositol-4,5-bisphosphate 3-kinase catalytic subunit alpha) result in abnormal activation of the phosphatidylinositol 3-kinase/protein kinase B/mammalian target of rapamycin (PI3K/Akt/mTOR, PAM) pathway, promoting tumor cell proliferation and therapeutic resistance, thereby necessitating additional PIK3CA inhibitor [5]. Moreover, the continuous clonal evolution of non-static tumor cells can generate new resistance mechanisms or cellular state alterations through epigenetic modifications. This high degree of complexity and sensitivity makes complete simulation extremely challenging.

Current biological observation methods are unable to simultaneously track all molecular events. High-throughput sequencing and high-resolution real-time imaging can only capture partial cellular cross-sections. This observational bottleneck limits the acquisition of comprehensive data required for constructing high-fidelity AI cell models under both perturbed and un-perturbed conditions. Advancing this field necessitates the development of more comprehensive cellular observation techniques, streamlined data collection approaches (given that biological data often contains substantial redundancy, leading to diminishing returns in model training), and more effective training methodologies.

Therefore, establishing cell-based AI learning models represents a more feasible approach, as making computational copies of actual cells may be impractical. This abstract model does not aim to fully simulate all cellular processes but instead focuses on learning from existing data to predict specific molecular or cellular phenotypes. AIVCs should be understood as an AI framework that serves as a structure for biological foundation models. This framework facilitates the connection and collaboration of various biological data, abstractly expressing and predicting cellular characteristics across multiple scales based on existing knowledge systems.

## Potential applications in oncology

### In silico experimentation reshaping cancer research paradigms

AIVC, emerging from the exponential growth of biological data, is poised to extend the methodological boundaries of traditional multi-omics experimental approaches. Using manipulator and decoder virtual instruments (VIs) that precisely regulate universal representations (URs) across different scales, AIVCs function as computational twins enabling top-down simulation and prediction. This capability is particularly valuable for cell types that are challenging to culture in vitro or molecular processes that are difficult to observe directly. For example, tissue-scale fine-tuned URs can replace classical single-cell resolution methods for identifying specific cell populations or functional domains within tumors. Given that the AIVC framework incorporates mechanisms for UR aggregation and transformation, representations from lower physical scales can generate representations at subsequent higher physical scales. This enables the interpretation of sub-tissue-scale phenotypes, such as dynamic cell–cell interactions, tumor evolution trajectories, and gene-epigenetic-protein signaling regulatory axes.

**Fig. 1:**
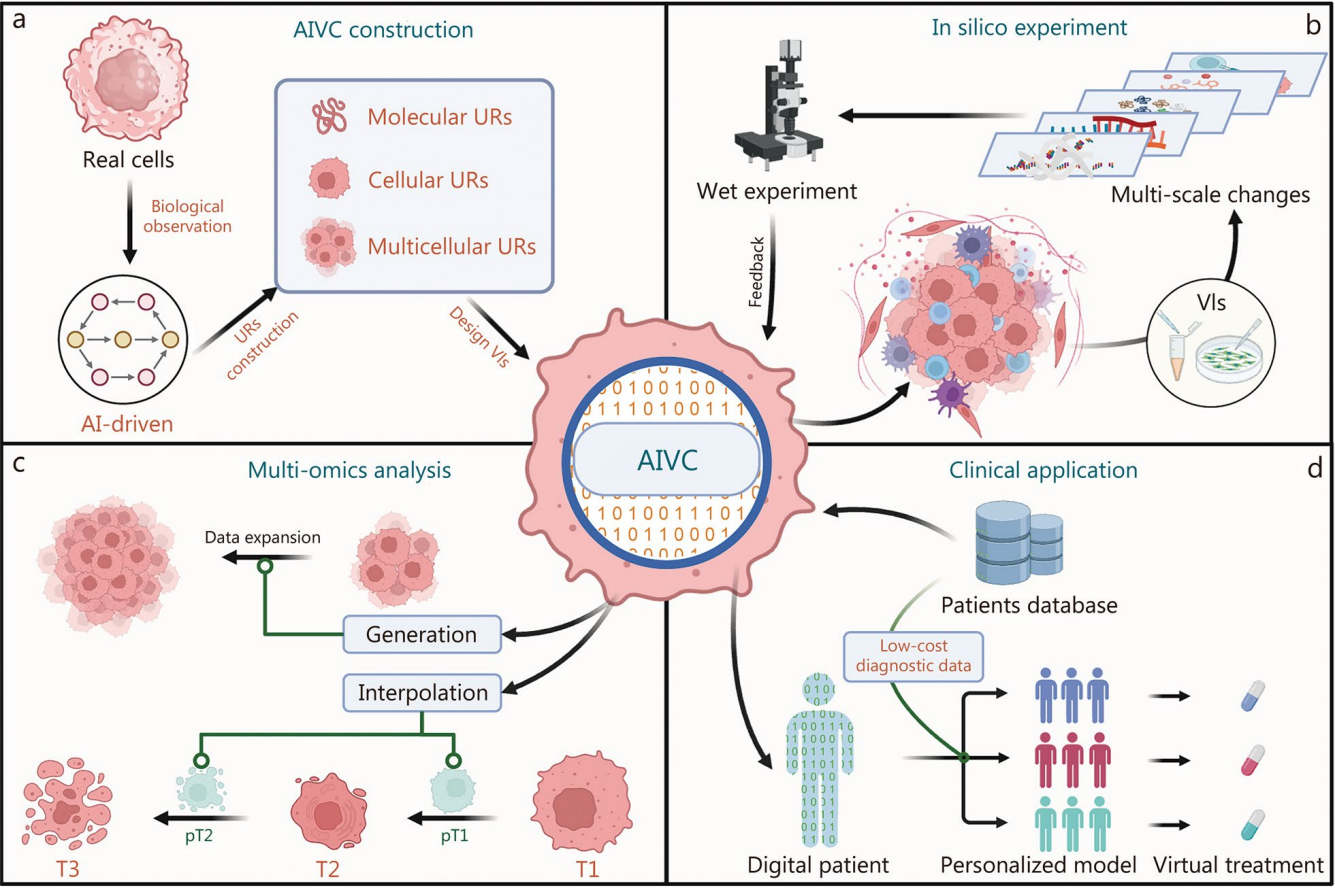
Potential applications in oncology. **a** Pattern diagram for building AIVCs using biological observation data. **b** In silico experimentation establishing new cancer research paradigms. By inputting specific tumor observational data, multi-scale tumor simulation can be achieved computationally. Integration with VIs enables virtual experiments and guides wet-lab experimentation, with feedback promoting continuous AIVCs iteration. **c** Enhancement of current tumor multi-omics analysis. By learning biological characteristics across numerous time points during tumor cell evolution, interpolation between discrete time points enables the reconstruction of tumor cell dynamic changes. Integration of generative AI methods into AIVCs expands scarce biological sample data. **d** Clinical applications. Standard digital patient models for specific diseases are constructed using extensive clinical diagnostic and treatment data. Individual patient models are created through novel low-cost diagnostic data, enabling virtual treatment simulations to predict efficacy and adverse reactions, better guiding actual drug administration. AIVCs artificial intelligence virtual cells, T1/2/3 actual process time points, pT1/2 AIVC-simulated pseudotime, URs universal representations, VIs virtual instruments

This would establish a new computational-experimental iterative research paradigm that provides predictive blueprints for translational and mechanistic studies of novel cancer therapeutics and treatment strategies: AIVCs perform large-scale computational screening of downstream effects and network reconstruction following treatments (such as drugs, cytokines, targeted inhibitors/activators), and provide specific experimental designs for validation and data generation. This is because the framework’s VIs, designed as specialized neural networks, can manipulate and interpret URs (Fig. 1a). When combined with perturbation experimental condition generative models and active learning mechanisms based on prediction confidence, AIVCs can conduct virtual experiments and provide results with confidence scores to guide subsequent experimental design while maintaining open data interfaces. Experimental data is then fed back into the system through data interfaces for further predictive analysis, thereby completing model iteration (Fig. 1b).

### Breaking through the bottleneck of multi-omics cancer research

The first breakthrough may address a fundamental challenge in multi-omics cancer research: reconstructing authentic dynamic changes in cellular states. Although continuous sampling can provide time-series data, discrete sampling methods remain inadequate for representing the authentic density of cellular state transitions. Furthermore, existing temporal analysis approaches infer developmental trajectories by ordering gene expression patterns of cell populations, which essentially represents a time-independent inference of cellular state progression. AIVCs address these dynamic challenges through a novel approach: by modeling the transient nature and continuous flux of cellular states, AI algorithms can interpolate between discrete time points to enable continuous simulation of molecular and cellular events. This facilitates more detailed observations of early tumorigenesis, where fine-tuning multi-scale URs allows for inferring the emergence of pre-malignant changes while capturing the influence of both genetic factors and environmental conditions (Fig. 1c).

By integrating generative AI methods, AIVCs can generate new data to support research on scarce biological samples, such as rare tumors or novel anti-cancer therapeutics [6]. These studies are typically constrained by limited sample sizes, forcing reliance on high-throughput sequencing combined with numerous low-throughput experiments. Generated data can be integrated into AIVCs, utilizing established thresholds to account for heterogeneity and homogeneity, thereby adjusting URs and interfacing with existing data to achieve effective data expansion. However, the reliability of generative AI content presents significant challenges. The ideal solution involves increasing the proportion of weak molecular interactions and rare tumor data during model construction while maintaining overall data balance. Given the complexity of modeling weak molecular interaction networks, this area still requires further technical breakthroughs and development (Fig. 1c).

### Accelerating anti-cancer drug development and personalized medicine

In translational medicine, AIVCs can serve as patient-specific digital models [7]. By integrating patient clinical information and tumor microenvironment characteristics to fine-tune AIVCs, disease-specific research platforms can be constructed. The primary advantage lies not in merely mapping relationships between patient outcomes and tumor characteristics to determine drug responses, but in simulating tumor cell behavior and microenvironmental changes as a high-throughput screening system for drug development. Although potentially less authentic than organoid models, combining virtual drug trials with organoid validation experiments ensures both screening efficiency and result reliability while accelerating drug development processes. Furthermore, updating models with novel low-cost diagnostic data such as circulating tumor DNA liquid biopsies [8] and radiomics [9], can generate individualized virtual models for each patient. This enables precise tumor diagnosis and personalized treatment, while also allowing virtual drug administration to patient digital models to preview potential treatment benefits and adverse reactions (Fig. 1d).

## Oncological problems in AIVC construction

### Evaluation: What matters most?

Model evaluation represents a critical challenge, as current biological foundation models lack unified assessment standards. Although these models generally demonstrate specific task simulation capabilities, their crucial generalization abilities, particularly essential for AIVCs, remain inadequately evaluated [1, 10]. Furthermore, heterogeneity is a defining characteristic that distinguishes cancer from other diseases. Even within the same type of tumor epithelial cells, differences in gene expression patterns due to genomic instability can lead to dramatically different treatment responses, and this heterogeneity continuously evolves throughout disease progression and treatment. Therefore, we propose that resolution power should be a key performance metric for evaluating models. Models must not only extract universal biological features across diseases and species from massive datasets but also maintain sufficient resolution to capture subtle heterogeneity characteristics. However, not all differences carry functional significance, and blindly increasing resolution can compromise the model’s ability to distinguish between technical noise, stochastic biological variation, and genuine physiological differences. AI engineers must strike a balance between these competing demands.

### Utilization of biological foundation models

Currently, numerous foundation models specifically target molecular and single-cell scales. Integrating these mature models with corresponding scale URs could accelerate the construction of AIVCs. However, model fusion strategies remain underdeveloped, presenting several critical challenges that require attention: model compatibility issues, disparities in input–output interfaces, considerations for specific module replacement, and selection of appropriate application scenarios. Moreover, during the integration process, establishing rigorous evaluation and benchmarking systems is essential to ensure that selected models align with research objectives while maintaining sufficient computational efficiency.

### Data imbalance challenges

Despite the unprecedented data explosion in bioinformatics, data distribution imbalance remains a crucial challenge in AIVC construction [1]. This imbalance manifests across multiple levels. For instance, the overrepresentation of high-incidence tumor types in databases leads to training data biases that may limit model generalization capabilities. At the molecular scale, data on molecular interactions exhibit significant bias. Current detection technologies often struggle to capture weak but biologically significant interactions. More critically, existing research disproportionately focuses on prominent molecular interactions, further exacerbating data imbalance. Additionally, significant variations in the proportions of different cell types within tissues result in inadequate characterization data for rare cell populations. Although efforts can be made to increase the proportion of scarce data, the overall scarcity remains a limitation, and its actual impact on specific tasks requires systematic evaluation.

## Outlook

The digitalization of life is a shared aspiration among scientists worldwide. Oncology, with its rapid development, is uniquely positioned to pioneer this digital transformation. While the proliferation of tumor omics databases and biological foundation models provides promising opportunities, significant challenges remain in establishing comprehensive AIVCs. Therefore, it is imperative to strengthen dialogue between clinicians, experimental scientists, and AI researchers to collectively advance cancer research and precision medicine, ultimately achieving the goal of conquering cancer.

## Data Availability

Not applicable.

## Abbreviations

AI: Artificial intelligence
AIVC: Artificial intelligence virtual cell
PAM: Phosphatidylinositol 3-kinase/protein kinase B/mammalian target of rapamycin (PI3K/Akt/mTOR)
URs: Universal representations
VIs: Virtual instruments

## References

[1] Bunne C, Roohani Y, Rosen Y, Gupta A, Zhang X, Roed M, et al. How to build the virtual cell with artificial intelligence: priorities and opportunities. Cell. 2024;187(25):7045–63. 39672099 10.1016/j.cell.2024.11.015 PMC12148494

[2] Yang T, Ma F, Qian H, Xu B. AI-driven construction of digital cell model. Innov Life. 2024;2(4):100102.

[3] Karr JR, Sanghvi JC, Macklin DN, Gutschow MV, Jacobs JM, Bolival B Jr, et al. A whole-cell computational model predicts phenotype from genotype. Cell. 2012;150(2):389–401. 22817898 10.1016/j.cell.2012.05.044 PMC3413483

[4] Maritan M, Autin L, Karr J, Covert MW, Olson AJ, Goodsell DS. Building structural models of a whole mycoplasma cell. J Mol Biol. 2022;434(2):167351. 34774566 10.1016/j.jmb.2021.167351 PMC8752489

[5] Turner NC, Im SA, Saura C, Juric D, Loibl S, Kalinsky K, et al. Inavolisib-based therapy in PIK3CA-mutated advanced breast cancer. N Engl J Med. 2024;391(17):1584–96. 39476340 10.1056/NEJMoa2404625

[6] Zitnik M, Li MM, Wells A, Glass K, Morselli Gysi D, Krishnan A, et al. Current and future directions in network biology. Bioinform Adv. 2024;4(1):vbae099. 39143982 10.1093/bioadv/vbae099 PMC11321866

[7] Katsoulakis E, Wang Q, Wu H, Shahriyari L, Fletcher R, Liu J, et al. Digital twins for health: a scoping review. NPJ Digit Med. 2024;7(1):77. 38519626 10.1038/s41746-024-01073-0 PMC10960047

[8] Moding EJ, Nabet BY, Alizadeh AA, Diehn M. Detecting liquid remnants of solid tumors: circulating tumor DNA minimal residual disease. Cancer Discov. 2021;11(12):2968–86. 34785539 10.1158/2159-8290.CD-21-0634 PMC8976700

[9] Zhang YP, Zhang XY, Cheng YT, Li B, Teng XZ, Zhang J, et al. Artificial intelligence-driven radiomics study in cancer: the role of feature engineering and modeling. Mil Med Res. 2023;10(1):22. 37189155 10.1186/s40779-023-00458-8 PMC10186733

[10] Liu J, Shen Z, He Y, Zhang X, Xu R, Yu H, et al. Towards out-of-distribution generalization: a survey. arXiv, 2021:2108.13624.

